# Depression and the Overall Burden of Painful Joints: An Examination among Individuals Undergoing Hip and Knee Replacement for Osteoarthritis

**DOI:** 10.1155/2015/327161

**Published:** 2015-03-11

**Authors:** Rajiv Gandhi, Michael G. Zywiel, Nizar N. Mahomed, Anthony V. Perruccio

**Affiliations:** ^1^Division of Orthopaedic Surgery, Toronto Western Hospital, University of Toronto, 399 Bathurst Street 1E449, Toronto, ON, Canada M5T 2S8; ^2^Institute of Health Policy, Management & Evaluation, University of Toronto, Toronto, ON, Canada

## Abstract

The majority of patients with hip or knee osteoarthritis (OA) report one or more symptomatic joints apart from the one targeted for surgical care. Therefore, the purpose of the present study was to investigate the association between the burden of multiple symptomatic joints and self-reported depression in patients awaiting joint replacement for OA. Four hundred and seventy-five patients at a single centre were evaluated. Patients self-reported joints that were painful and/or symptomatic most days of the previous month on a homunculus, with nearly one-third of the sample reporting 6 or more painful joints. The prevalence of depression was 12.2% (58/475). When adjusted for age, sex, education level, hip or knee OA, body mass index, chronic condition count, and joint-specific WOMAC scores, each additional symptomatic joint was associated with a 19% increased odds (odds ratio: 1.19 (95% CI: 1.08, 1.31, *P* < 0.01)) of self-reported depression. Individuals reporting 6 or more painful joints had 2.5-fold or greater odds of depression when compared to those patients whose symptoms were limited to the surgical joint. A focus on the surgical joint alone is likely to miss a potentially important determinant of postsurgical patient-reported outcomes in patients undergoing hip or knee replacement.

## 1. Introduction

Both depression and generally poorer mental well-being are more prevalent among individuals with chronic medical conditions when compared to those without chronic disease [1, 2]. Additionally, poorer mental health status has been consistently shown to be associated with reduced overall health-related quality of life and with poorer outcomes of medical and/or surgical treatments [3, 4]. Investigators have reported that pain associated with a range of chronic physical conditions is a significant contributing factor to increased rates of depression [5, 6].

Osteoarthritis (OA) is one of the most prevalent chronic medical conditions globally and is a leading cause of disability and health care utilization [7]. The cardinal symptom of OA, as well as a leading factor for seeking medical care and surgical intervention, is pain [8]. Chronic pain is a strong predictor of clinical depression in this population, and both factors contribute to patient-reported functional disability associated with OA [9, 10]. A number of studies have investigated the occurrence of depressed mood among OA clinical populations. One study, for instance, reported a 20% prevalence of depressive mood associated with OA within a primary care population [10], and similar rates have been documented among OA populations seeking specialist care [11, 12]. Depressed mood has been linked with poorer functional outcomes of nonsurgical care in longitudinal, observational studies of knee OA [4] and with poorer pain and function scores and lower satisfaction, following interventions such as hip and knee replacement surgery for OA [3, 13, 14]. While several studies have examined the relationship between depressed mood and pain in OA and a number of studies have reported worse pain to be associated with more severe depression scores [10], the vast majority of investigations to date limited pain assessments to a single joint of interest. However, individuals with OA in one joint will often have symptomatic disease in one or more other joints, resulting in variable symptomatic disease burden that may not be appreciated when assessments are limited to a single joint [15–17].

Therefore, the purpose of this study was to investigate the association between the overall burden of symptomatic joints and self-reported depression among patients awaiting hip or knee replacement for end stage OA. We hypothesized that those with a greater burden of painful joints would report a greater prevalence of depression, independent of the magnitude of hip- or knee-specific pain in the surgical joint.

## 2. Methods

Patients with end stage hip or knee OA scheduled to undergo elective, primary total joint replacement were consecutively recruited from an academic hospital in Toronto, Canada, from April 2010 to November 2012. Eligibility criteria limited participants to those 18 years of age or older at the time of assessment and those who were able to read and understand English. Individuals with inflammatory arthritis or posttraumatic OA were excluded. Participants were mailed a health questionnaire consisting of a series of demographic and clinical questions and were asked to complete it two weeks prior to their scheduled surgical date. The study was approved by the University Health Network Research Ethics Board, and written informed consent was obtained from all study participants.

Demographic characteristics collected included age, sex, and level of education (≤secondary school, >secondary school). Height and weight were abstracted from the clinical record of the preadmission assessment and used to calculate body mass index (BMI) in kg/m^2^. The type of procedure planned (hip or knee replacement) was recorded for use as an indicator variable in the analyses described below.

Self-reported clinical data collected using the health questionnaire included medical comorbidities, number and location of symptomatic joints, and surgical joint-specific pain and function.

Individuals were asked to respond with yes or no to a series of questions asking whether they had one or more of a number of medical conditions. Additionally, for each question answered in the affirmative, patients were asked whether they were currently undergoing treatment for that condition [18]. Patient answers to the question of whether they had depression were used as the primary outcome of interest.

The number of locations of symptomatic joints was collected using a homunculus, on which participants were asked to graphically indicate all joints that had been painful/symptomatic for most days of the previous month. The graphical responses were examined and divided into discrete anatomical regions including neck, spine, shoulders, elbows, wrists, hands, hips, knees, ankles, and feet, with laterality noted as appropriate. Any marked joint was considered a symptomatic joint, consistent with the clinical criteria of the American College of Rheumatology [19, 20]. The number of joints indicated were summed to derive a painful joint score (ranging from 1 to 20), which formed the primary predictor of interest. The assessment was limited to joint pain only; no attempt was made to register the presence and/or distribution of more widespread, nonarticular, and/or myofascial pain. Similarly, patients were not specifically queried about the presence or absence of medical diagnoses such as fibromyalgia, which are characterized by widespread, nonarticular pain patterns.

Hip- or knee-specific pain and function was assessed using the Western Ontario McMaster University Osteoarthritis Index (WOMAC) pain and function subscales, respectively. The WOMAC index is among the most commonly used pain and function measures in lower-extremity OA populations, with demonstrated validity and responsiveness [21, 22]. Possible scores range from 0 to 68 for the functional limitation scale and 0 to 20 for the pain scale, with higher scores indicating greater limitations/pain.

### 2.1. Statistical Analyses

Descriptive statistics were obtained for the overall sample. Bivariable analyses were performed to compare the distribution of variables between individuals who did and did not report depression using either the independent samples *t*-test or chi-square test, as appropriate.

Logistic regression modeling was used to investigate the association between painful joint count and reported depression (outcome), adjusted for age, sex (reference: female), level of education (reference: >high school), hip/knee OA group (reference: hip), BMI, comorbidity count, and WOMAC hip/knee-specific pain and functional limitation scores.

## 3. Results 

Five hundred fifty-nine (559) individuals were eligible and enrolled in the study. Of these, 45 (8%) withdrew from the study prior to returning the questionnaire or did not complete their questionnaires for other reasons. Five hundred and fourteen patients returned their questionnaires, of which 475 had complete data (207 hip, 268 knee). In total, data concerning 85% of eligible patients (475/559) were included in the final analysis.

The patients in the final study cohort had a mean age of 64.7 years, 57% of whom were female. The overall prevalence of patient-reported depression was 12.2% (58/475), with 91% of those patients (53/58) further reporting receiving treatment for this diagnosis at the time of questionnaire completion. Patients reporting depression were significantly more likely to be female, to have a higher BMI, higher comorbidity counts, and higher painful joint count (Table 1).

When adjusting for covariates using logistic regression modeling, an independent association was identified between the number of symptomatic joints and the likelihood of reporting depression (Table 2). Each numeric increase in symptomatic joint count was associated with 19% increased odds of reporting depression (odds ratio: 1.19 (95% CI: 1.08, 1.31, *P* < 0.01)) (Table 2).

## 4. Discussion

Depression has been associated with poorer treatment adherence in general clinical populations [23] and with worse health-related outcomes following both surgical and nonsurgical treatment in patients with OA specifically [3, 4]. As a result, an improved understanding of factors that contribute to depression in patients with OA and the identification of potentially modifiable correlates of depression may allow for the development of tailored treatment strategies that will positively impact outcomes in these patients. Despite the common presence of multiple symptomatic joints among individuals with OA in any one joint, studies to date have commonly limited their clinical assessment to the single joint of interest exclusively. It is likely that physicians and surgeons frequently similarly limit their clinical assessments as well. However, the results of the present study demonstrate that the presence of multiple other symptomatic joints in patients with end stage hip and knee OA is strongly associated with patient-reported depression.

Having been adjusted for several relevant factors, including age, sex, and other comorbid conditions, each numeric increase in painful joint count increased the odds of patient-reported depression by almost 20%. It is important to recognize that this relationship is cumulative and that in the present study patients could have up to 20 painful joints. Thus, individuals reporting 6 or more painful joints (encompassing one-third of the sample) had at least 2.5-fold increased odds of reporting depression compared to those who had symptoms in the surgical joint only. These findings speak to the importance of adopting a more holistic approach to the assessment of individuals with OA in the context of both clinical and surgical care and research activities [16]. While not specifically assessed in the present study, depression and depressed mood have been consistently shown to predict poorer patient-reported outcomes, including pain, activity limitations, and satisfaction with surgery [13, 14]. Thus, the present results suggest that patients with OA and multiple painful joints may benefit from aggressive symptom management and potential psychological assessment [24].

Our findings are consistent with previous studies that have reported multisite body pain to be associated with incident depression among general medical and surgical populations [25, 26]. A range of mediating links between chronic pain and depression have been proposed, ranging from altered neurotransmitter activity (norepinephrine, serotonin) within the central nervous system [27] to chronic sleep problems (chronic insomnia, unrefreshing sleep) [28]. The experience of chronic pain is reported to provoke a range of psychological reactions including helplessness, anxiety, depression, and anger [27]. Sleep-interrupting pain increases fatigue, while social relationships with friends and family may be adversely affected as well [28–30]. However, while depression, anxiety, and negative affect have been previously reported to be associated with magnitude of OA pain [31, 32], the causal direction of such relationships is difficult to discern. Unfortunately, the cross-sectional design of the present study prevents any assessment of directionality.

We acknowledge several potential limitations with the present study. First, our study sample was limited to individuals with late stage hip or knee OA associated with chronic joint pain. Second, while a longer duration of pain has been associated with a greater likelihood of depression [33], the duration of symptoms in the nonsurgical joints was not assessed in the patient questionnaire and thus was not considered in the analyses. Third, several factors may have influenced the prevalence of depression in the present study (12%), which was lower than that reported in some other OA patient populations (20%). Surgical candidacy is based on a number of factors that include medical fitness, and thus the present study cohort may not be representative of all OA patient populations. Furthermore, while many studies to date have stratified patients based on depressive symptoms identified using self-reported mood questionnaires, we defined depression as a self-reported condition without any attempt to confirm the presence of diagnostic criteria for this condition or any assessment of its chronicity. Nevertheless, with 91% of patients who self-reported having depression in the present study also reporting current treatment for it, it would appear that the large majority carried the formal diagnosis typically required before starting treatment. However, because the present study relied on self-reported depression, it is possible that some cases were missed because of imprecise recollection, unwillingness to report, or by a lack of clinical diagnosis. The extent to which missed cases relate to degree of painful joint burden is unknown. Fourth, 15% of eligible patients either withdrew from the study or did not complete their questionnaire data. The lack of data for these patients precluded comparison with those who remained in the study, and thus the possibility of selection bias cannot be excluded. Fifth, no data were available concerning the presence and/or severity of widespread nonarticular pain patterns or the presence of central pain conditions such as fibromyalgia. Patients with fibromyalgia are known to sometimes report joint pain, and this condition has also been reported to be associated with poorer mental well-being. Thus, while the relationship between multiple symptomatic joints and central pain conditions in patients with known OA is not well understood, some of the findings of the present study may be explained by the concomitant presence of fibromyalgia or other similar conditions. As such, it would seem to be important to screen patient with OA for the presence of a comorbid central pain condition. Unfortunately, insufficient data were available to explore these relationships in the present study. Nevertheless, despite these limitations, we feel that the results of the present study do provide novel evidence for a clinically relevant association between the symptomatic joint burden in patients with OA and self-reported depression.

## 5. Conclusions

Given our finding that the presence of multiple painful joints in patients with established hip or knee OA is associated with an increased risk of prevalent depression, as well as likely depressed mood, an exclusive focus on the surgical joint alone is likely to miss a potentially important determinant of postsurgical patient-reported outcomes in this population. Consistent with other studies, we report that multiple painful joints are frequent in this population. While further study is needed to better understand the specific reasons for these associations, the authors suggest that clinicians and researchers consider the development of tailored treatment and management strategies for OA based on degree of multiple joint involvement, along with patient education, to address the unique characteristics of this distinct subgroup of patients.

## Figures and Tables

**Table 1:** Comparison of demographic and clinical data between patients who did and did not report depression.

	Depression = yes	Depression = no	*P* value
	(*n* = 58)	(*n* = 417)	
Mean age and SD (range)	62.7 ± 9.9 (37 to 86)	65.0 ± 10.1 (33 to 89)	0.105
Percentage of female patients	82.5	53.5	<0.001
Percentage of patients without any formal education beyond high school	27.6	30.8	0.618
Percentage of patients awaiting knee (versus hip) replacement	62.1	55.6	0.355
Mean body mass index in kg/m^2^ and SD (range)	33.5 ± 8.7 (21 to 62)	29.9 ± 6.3 (19 to 55)	<0.001
Mean number of comorbidities and SD (range)	2.7 ± 1.7 (0 to 7)	1.7 ± 1.3 (0 to 7)	<0.001
Mean WOMAC pain score and SD in points (range)	10.2 ± 4.2 (0 to 20)	9.6 ± 3.7 (1 to 18)	0.238
Mean WOMAC function score and SD in points (range)	34.7 ± 13.7 (0 to 68)	31.6 ± 12.1 (0 to 62)	0.073
Mean number of painful joints and SD (range)	7.3 ± 4.7 (1 to 20)	4.1 ± 3.1 (1 to 18)	<0.001

SD: standard deviation; WOMAC: Western Ontario and McMaster Universities Osteoarthritis Index.

**Table 2:** Results of logistic regression evaluating associations between symptomatic joint count and depression.

Predictor variable	Reference value	Contrast value	Odds ratio	95% confidence interval
Age in years	—	—	**0.96**	**0.93, 0.99**
Sex	Male	Female	**4.24**	**1.74, 10.35**
Education	Some formal education beyond high school	No formal education beyond high school	1.05	0.50, 2.23
Surgical joint	Hip	Knee	0.70	0.34, 1.45
Body mass index	—	—	1.04	0.99, 1.09
Number of comorbidities	—	—	**1.50**	**1.15, 1.85**
WOMAC pain score	—	—	0.89	0.76, 1.05
WOMAC function score	—	—	1.02	0.97, 1.07
Number of symptomatic joints	—	—	**1.19**	**1.08, 1.31**

WOMAC: Western Ontario and McMaster Universities Osteoarthritis Index.

## Abbreviations

OA: Osteoarthritis
WOMAC: Western Ontario and McMaster Universities Osteoarthritis Index.
